# Vitamin D deficiency in children with acute bronchiolitis: a prospective cross-sectional case- control study

**DOI:** 10.1186/s12887-021-02666-4

**Published:** 2021-04-30

**Authors:** Inbal Golan-Tripto, Neta Loewenthal, Asher Tal, Yotam Dizitzer, Yael Baumfeld, Aviv Goldbart

**Affiliations:** 1 Department of Pediatrics, Soroka University Medical Center, Beer-Sheva, Israel; 2 Pediatric Pulmonary Unit, Soroka University Medical Center, Beer-Sheva, Israel; 3 Clinical Research Center, Soroka University Medical Center, Beer Sheva, Israel; 4 Faculty of Health Sciences, Ben-Gurion University of the Negev, PO box 151, Beer-Sheva, Israel; 5 Pediatric Endocrinology Unit, Soroka University Medical Center, Beer-Sheva, Israel

**Keywords:** Bronchiolitis, Vitamin D deficiency, Vitamin D status, Children

## Abstract

**Background:**

25(OH) vitamin D levels are inversely associated with respiratory infections and childhood wheezing.

**Objective:**

To evaluate serum 25(OH) vitamin D levels in infants and toddlers with acute bronchiolitis, compared to subjects with non-respiratory febrile illness*.*

**Methods:**

A prospective cross-sectional case-control study which compared serum 25(OH) vitamin D levels between infants and toddlers diagnosed with acute bronchiolitis to subjects with non-respiratory febrile illness. Multivariate logistic regression, adjusted for age, sex, ethnicity and nutrition was performed. Correlation between serum vitamin D levels and bronchiolitis severity was assessed via Modified Tal Score and length of hospital stay (LOS).

**Results:**

One hundred twenty-seven patients aged < 24 months were recruited; 80 diagnosed with acute bronchiolitis and 47 patients with non- respiratory febrile illnesses. Both groups had similar demographics aside from age (median [IQR] 5 [3–9] vs. 9 [5–16] months in the bronchiolitis group compared to control group (*p* = 0.002)). Serum 25(OH) vitamin D levels were significantly lower in the bronchiolitis group; median [IQR] 28[18–52] vs. 50[25–79] nmol/L, respectively, (*p* = 0.005). Deficient vitamin D levels (< 50 nmol/L) was found more frequently in the bronchiolitis group than controls; 73% vs. 51% (*p* = 0.028). Multivariate logistic regression showed vitamin D deficiency was more probable in bronchiolitis patients; OR [95% CI] 3.139[1.369–7.195]. No correlation was found between serum vitamin D levels and bronchiolitis severity, which was assessed via Modified Tal Score and by length of hospital stay.

**Conclusion:**

Children with acute bronchiolitis displayed significantly lower vitamin D levels than children with non-respiratory acute febrile illnesses.

## Background

Acute bronchiolitis is a major cause of morbidity during infancy and early childhood. The leading pathogen of acute bronchiolitis is respiratory syncytial virus (RSV) [1]. The importance of vitamin D is well described; it plays a key role in the activation of the innate immune system [2], particularly during lower respiratory tract infections [3–5]. Vitamin D increases mucociliary clearance, regulate epithelial cell production and modulate inflammatory pathways [6].

Previous studies have suggested that cord 25(OH) vitamin D levels are significantly and inversely associated to the prevalence of respiratory infections and childhood wheezing [7].Furthermore, vitamin D deficiency was associated with acute bronchiolitis [8, 9], poor asthma control [10], severe asthma exacerbations [11], and high consumption of asthma medications. To understand the role of vitamin D in acute bronchiolitis further, we examined serum levels of 25(OH) vitamin D in infants younger than 24 months, that were clinically diagnosed with acute bronchiolitis. We compared those patients to a control group of patients that had non-respiratory febrile illnesses such as urinary tract infection, acute gastroenteritis and dysentery. We hypothesized that children with acute respiratory illness, such as acute bronchiolitis, will have lower vitamin D levels, since vitamin D and its receptor (VDR) were found in the epithelial cells of respiratory tract and influence mucocilliary clearness. We also investigated whether 25(OH) vitamin D levels were correlated with bronchiolitis severity, measured with the Modified Tal Score (MTS) [12] or with the length of hospital stay (LOS).

## Methods

### Setting, subjects and data

This prospective cross-sectional case-control study was conducted at Soroka University Medical Center (SUMC), the single tertiary medical center in Southern Israel, with a catchment population of approximately 750,000. The Division of Pediatrics in SUMC contains 100 hospital beds with an annual activity of 35,000 ED (emergency department) visits and 12,000 hospitalizations; within them, approximately 400 admissions in three pediatric departments due to acute bronchiolitis. We recruited during winter season (December to March) in one of the pediatric departments.

Infants and toddlers 24 months old and younger, who were referred to the ED at SUMC with febrile illness, were invited to participate in our study. Children whose legal caregivers signed an informed consent form and who met none of the exclusion criteria were recruited. Exclusion criteria were chronic diseases that could influence the severity and course of bronchiolitis and included: chronic lung disease, congenital heart disease, inborn errors of metabolism, psychomotor retardation, hypotonia or any muscular abnormalities.

Upon clinical evaluation and initial treatment in the ED, children who were diagnosed with acute bronchiolitis were defined as cases. Children 24 months and younger with acute febrile illness (fever > 38.5°c, duration > 24 h) but without respiratory symptoms, were defined as controls.

Bronchiolitis patients underwent bronchiolitis severity assessment in the ED setting using the MTS. Bronchiolitis severity was defined by the following: scores ≤5, mild; scores 6 to 10, moderate; and scores ≥11, severe. Bronchiolitis patients also underwent a nasal wash to assess respiratory viral antigens.

Questionnaires regarding demographical data, past medical history, Vitamin D administration, risk factors for asthma and Vitamin D deficiency and family history, were filled for all patients upon referral to the ER. Blood samples for Vitamin D serum levels were obtained.

Participants were followed throughout their clinical course of treatment and clinical data was recorded and documented. Collected data included whether patients were discharged from the ER or admitted to the hospital, length of stay upon hospitalization, admission to the pediatric intensive care unit (PICU) and need for mechanical ventilation.

All methods in the study were carried out in accordance with the Helsinki guidelines and declaration or any other relevant guidelines. The study was approved and overseen by the Institutional Review Board Committee of SUMC (Number- 5122).

### Study outcomes

The primary outcome was the level of serum 25(OH) Vitamin D compared between bronchiolitis and non-bronchiolitis patients. Secondary outcome was the association between disease severity and serum Vitamin D levels within bronchiolitis. Disease severity was assessed by length of stay upon admission to the hospital for all patients and bronchiolitis severity assessment via MTS for bronchiolitis patients only.

### Laboratory measurements

Blood samples were drawn from all subjects upon admission. Samples were refrigerated at 4 °C and then centrifuged within 24 h. The serum was stored at − 80 °C until analysis. Levels of 25(OH) vitamin D were measured in duplicate, with the Diasorin chemiluminescence immunoassay. We categorized 25(OH) vitamin D levels as: deficient: < 50 nmol/L: insufficient: ≥50 and < 75 nmol/L; and sufficient: ≥75 nmol/L. We performed a nasal wash for participants of the bronchiolitis group to recover respiratory viral antigens, which were assessed with PCR.

### Statistical analysis

We described and compared demographical and clinical characteristics and serum vitamin 25(OH) D levels between cases and controls. Variables were described as follows - continuous variables with normal distribution as mean ± SD, continuous variables with non-normal distribution or ordinal variables as median and interquartile range (IQR) and categorical data as sum and percentage. Univariate comparison between groups was performed with appropriate tests. Specifically, nominal variables were compared via Pearson’s chi-square test; continuous variables that fulfilled parametric criteria were compared via Student’s t-test; and ordinal variables and continuous non-parametric variables were compared via Wilcoxon or Mann-Whitney U tests.

Furthermore, we designed a multivariate logistic regression model to assess the risk of vitamin D deficiency in the bronchiolitis patients, compared to controls, adjusted to age, sex, ethnicity and means of nutrition (whether they were breast fed, formula fed or both). Results are presented as Odds Ratio (OR) with 95% confidence interval (CI) and depicted via forest plot graph.

Third, we evaluated correlations between serum 25(OH) vitamin D levels and LOS upon admission, for all hospitalized patients. For bronchiolitis patients only we evaluated correlations between serum 25(OH) vitamin D levels and bronchiolitis severity (assessed by MTS severity score). Correlation was calculated via Pearson’s chi square for parametric variables and via Spearman’s correlation coefficient for non-parametric variables.

For all analyses, two-tailed *p*-values ≤ .05 were considered statistically significant. Data were analyzed with IBM SPSS© statistics software package version 23.

## Results

### Patient characteristics

A total of 127 participants were recruited, including 80 (63%) in the bronchiolitis group and 47 (37%) in the control group (febrile, non-bronchiolitis). The groups had similar demographic details (Table 1) aside from age. Bronchiolitis patients were shown to be younger than febrile non-bronchiolitis patients; median age of 5 months versus 9 months, respectively (p 0.002). The control group consisted of children with fever that were diagnosed with: acute gastroenteritis (*n* = 12, 26%), acute otitis media (*n* = 8, 17%) urinary tract infection (*n* = 4, 9%), clinical dysentery (*n* = 4, 9%), occult bacteremia (*n* = 5, 11%), cellulitis/ skin infection (*n* = 2, 4%) neonatal fever (*n* = 1, 2%) and nonspecific viral infection (*n* = 10, 21%). More than half of the subjects in the bronchiolitis (59%) and control (51%) groups were of Bedouin origin. According to the guidelines of the Israeli Health Ministry, children under the age of 1 year are recommended to be treated with supplementary vitamin D, 400 IU per day. However, only 66% of the bronchiolitis group and 60% of the control group received vitamin D. Nasal washes for viral antigen were performed in 72 of 80 bronchiolitis diagnosed subjects (90%). Among these 72 subjects, we detected RSV in 71% (*n* = 51), human metapneumo virus in 11% (*n* = 8), influenza A virus in 8% (*n* = 6), adenovirus in 3% (*n* = 2), one RSV & corona virus co-infection (*n* = 1), one RSV& influenza A virus co-infection (*n* = 1), and negative results from a respiratory viral panel in 7% (*n* = 5).

**Table 1:** Demographic and clinical characteristics of infants in the bronchiolitis and control groups

Characteristic	Bronchiolitis Group *N* = 80	Control Group *N* = 47	*p* value
Demographic charachteristics			
Age, months ^a^	5 [3–9]	9 [5–16]	0.002
Sex (male) ^b^	47 (58%)	23 (49%)	0.283
Ethnicity (Bedouin) ^b^	47 (59%)	24 (51%)	0.400
Attend day care ^b^	21 (28%)	16 (34%)	0.451
Medical history and clinical characteristics			
Prematurity (≤ 37 weeks gestation) ^b^	9 (11%)	5 (11%)	0.915
Nutrition ^b^:			
Exclusive breast feeding (until 4 mo.)	22 (28%)	13 (25%)	0.619
Formula	18 (23%)	16 (31%)	
Breast feeding and formula	38 (49%)	23 (44%)	
Vitamin D administration ^b^	53 (66%)	28 (60%)	0.450
Passive exposure to smoking ^b^	27 (37%)	17 (36%)	0.981
1st degree relative with asthma ^b^	36 (47%)	11 (23%)	0.008
Allergic rhinitis ^b^	5 (6%)	1 (2%)	0.290
Atopic dermatitis ^b^	3 (4%)	2 (4%)	0.888
Cow’s milk protein allergy ^b^	2 (3%)	4 (9%)	0.123
Previous respiratory episode ^b^	23 (29%)	15 (32%)	0.707
Previous systemic steroid treatment ^b^	18 (23%)	11 (23%)	0.907
Previous treatment with beta agonists ^b^	26 (33%)	15 (32%)	0.946
Hospitalization associated characteristics			
Length of stay (days) ^a^	4 [2–6]	1 [0–3]	< 0.001

Table 1 shows population characteristics including demographic characteristics, medical history and clinical characteristics. Univariate analysis was held to assess the difference between bronchiolitis vs. control groups. ^a^variables are depicted by Median and [IQR] and compared via Man-Whitney U test ^b^variables are depicted by n(%) and compared via Pearson’s Chi-square;. Statistical significance is represented by *p* value

Regarding clinical characteristics of admitted patients, bronchiolitis patients had a significantly longer LOS than non-bronchiolitis; median of 4 days and 1 day, respectively (*p* < 0.001).

### Bronchiolitis and vitamin D deficiency

Serum 25(OH) vitamin D levels were lower in the bronchiolitis group; median [IQR] 28 [18–52] nmol/L and 50 [25–79] nmol/L, respectively, p 0.005. Deficient 25(OH) vitamin D levels (< 50 nmol/L) were found more frequently in the bronchiolitis group than in the control group and sufficient 25(OH) vitamin D levels were found more frequently in the control group than in the bronchiolitis group (Table 2).

**Table 2:** Serum 25(OH) vitamin D levels in infants in the bronchiolitis and control groups

	Bronchiolitis Group *N* = 80	Control Group *N* = 47	*p* value
Serum Vitamin D levels (nmol/L) ^c^	28 [18–52]	50 [25–79]	0.005
Vitamin D catagories ^b^:			
Deficient (Vitamin D: ≤ 50 nmol/L)	58 (73%)	24 (51%)	0.028
Insufficient (Vitamin D: 50–75 nmol/L)	13 (16%)	10 (21%)	
Sufficient (Vitamin D: ≥ 75 nmol/L)	9 (11%)	13 (28%)	

Table 2 shows levels of serum vitamin D and vitamin D categories. Univariate analysis was held to assess the difference between bronchiolitis vs. control groups. variables are depicted by Mean ± SD and compared via Students’ T-test; ^b^variables are depicted by n(%) and compared via Pearson’s Chi-square; ^c^variables are depicted by Median and [IQR] and compared via Man-Whitney U test. Statistical significance is represented by *p* value

**Fig. 1:**
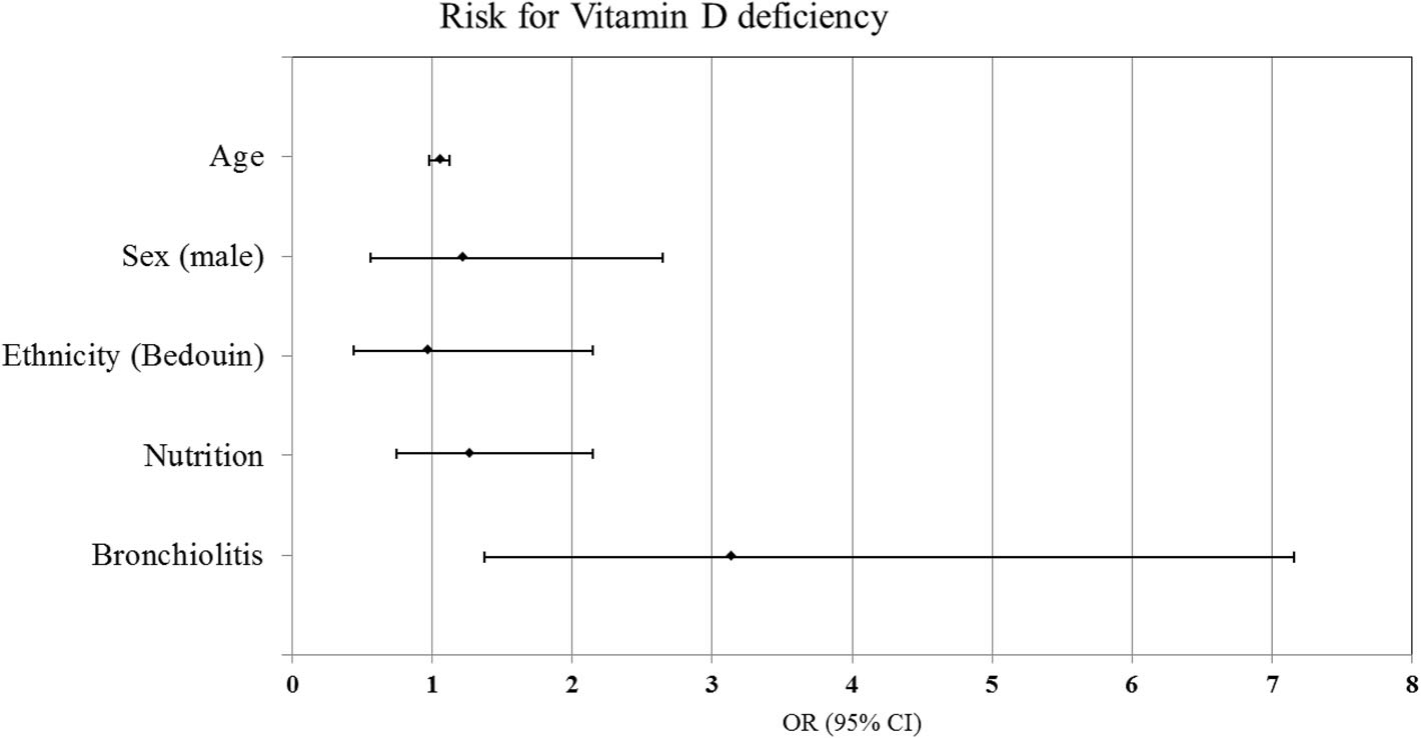
Forest plot for multivariate logistic regression assessing Odds Ratio for Vitamin D deficiency among the bronchiolitis and control group adjusted for age, sex, ethnicity and means of nutrition. Results are presented as odds ratio (OR) with 95% confidence interval (CI)

Multivariate logistic regression, adjusted for age, sex, ethnicity and nutrition, showed that bronchiolitis patients were more likely to have Vitamin D deficiency compared to febrile non-bronchiolitis patients; OR [95% CI] 3.139 [1.369–7.195] (Fig. 1).

**Fig. 2:**
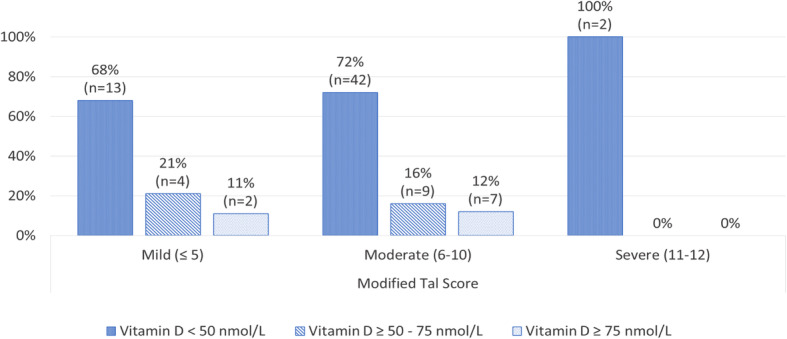
Different 25(OH) vitamin D levels within grouped modified Tal score in the bronchiolitis group (*n* = 79)

**Fig. 3:**
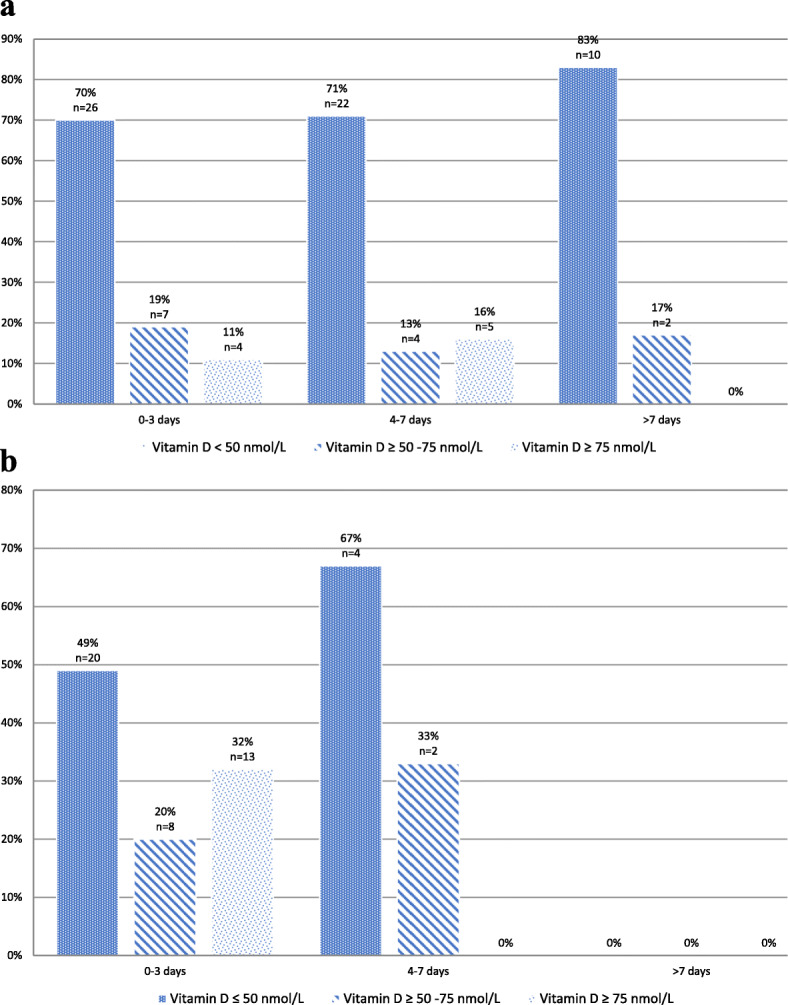
**a** Different 25(OH) vitamin D levels, grouped by length of stay in bronchiolitis group (*n* = 79). **b** Different 25(OH) vitamin D levels, grouped by length of stay in non-bronchiolitis group (*n* = 47)

For the bronchiolitis group, no statistically significant correlation was found between serum vitamin D levels and bronchiolitis severity via MTS; Spearman’s rho − 0.189, p 0.095. However, bronchiolitis patients showed higher percentages of children with vitamin D deficiency divided by levels of clinical severity depicted by MTS (Fig. 2). For bronchiolitis and non-bronchiolitis patients, no statistically significant correlation was found between serum Vitamin D levels and LOS; Spearman’s rho − 0.143, p 0.204 and 0.052, p 0.726, respectively. However, bronchiolitis and non-bronchiolitis patients both showed higher percentages of serum Vitamin D deficiency within patients with longer LOS (Fig. 3a and b, respectively).

## Disscussion

Our results demonstrated that vitamin D deficiency was associated with acute bronchiolitis, compared to febrile non-bronchiolitis illnesses, when adjusted to age, sex, ethnicity and means of nutrition. Previous studies have reported inconsistent findings regarding the role of vitamin D status in acute bronchiolitis.

A potential explanation for why a vitamin D deficiency was associated with an acute respiratory tract infection might be related to its important role in the innate immune system. Vitamin D is involved in inducing the activity of the endogenic antimicrobial proteins, defensins and cathelicidin, which fight against bacterial and viral infections [13, 14] in bronchial epithelial cells [15]. Wang et al. [16] showed that vitamin D (1,25(OH)_2_D_3_) could directly induce the cathelicidin antimicrobial peptide and defensin β_2_, which suggested that it could provide enhanced antimicrobial protection against infections. This protection accelerated healing after surgery. Janssen et al. [17] demonstrated a genetic association between polymorphisms in the vitamin D receptor and the severity of RSV bronchiolitis [5]. Other experimental studies have indicated that both ultraviolet B-ray exposure and oral vitamin D supplementation could raise the level of cathelicidin in skin [18]. It is known that vitamin D status is determined largely from ultraviolet B-ray exposure; therefore, 25(OH) vitamin D levels are lower during winter, the peak season for acute bronchiolitis. Both groups were recruited during winter, thus eliminating season as a factor that influence vitamin D levels. Taken together, these clinical and mechanistic data supported our hypothesis that vitamin D might be an important modulator of the immune response to respiratory viruses in acute bronchiolitis. Moreover, our control group had acute infections, such as acute gastroenteritis or urinary tract infections, without respiratory symptoms. Thus, our findings suggested that vitamin D status played a specific protective role in respiratory tract infections, but not necessarily in other infections. On the other hand, Thornton et al. reported that vitamin D deficiency was associated with increased rates of diarrhea with vomiting and with earache and/or discharge with fever, but was not significantly related to cough with fever in school-age children [19].Different findings were brought up by Hassam et al. [20] .that did not find association between vitamin D levels and diarrhea in children under 5 years of age.

We could not demonstrate a correlation between 25(OH) vitamin D levels and bronchiolitis severity (estimated with the MTS severity score) or LOS. Similar results were reported by Beigelman et al. [21], who studied 145 infants and concluded that the vitamin D status during acute bronchiolitis was not associated with indicators of bronchiolitis severity. Roth et al. [22] studied 120 hospitalized Canadian children under 2 years old, and found no association between vitamin D status and acute lower respiratory infections.

A potential explanation for this association might be that vitamin D can shift immunomodulatory effects towards T helper cell (Th)-2 responses [23]. In contrast, RSV bronchiolitis severity is inversely proportional to the Th-1 response [24]. This was observed when RSV was the causing pathogen. On the other hand, in a previous study on 28 Japanese children, more children with lower respiratory tract infection that required supplementary oxygen and ventilator management were also 25(OH) vitamin D deficient [25]. McNally reported that the mean vitamin D level was significantly lower in subjects with acute lower respiratory tract infections admitted to the pediatric intensive care unit, compared to children admitted to the general pediatrics ward [26]. That finding implied that vitamin D might be associated with severe respiratory illnesses. Indeed, among our 80 subjects with bronchiolitis, those admitted to the pediatric intensive care unit and those with prolonged hospitalizations had the lowest 25(OH) vitamin D levels. Moreover, we would like to emphasize that the 25(OH) vitamin D levels were taken upon admission; thus, the level was not influenced by the LOS.

Although we could not demonstrate a correlation between bronchiolitis severity and 25(OH) vitamin D deficiency, we found that two subjects with severe bronchiolitis (i.e., those with the MTS ≥ 11) or subjects with the longest hospitalizations had insufficient levels of 25(OH) vitamin D. Therefore, vitamin D could serve as a marker of severity. The correlation between 25(OH) vitamin D deficiency and bronchiolitis severity was demonstrated in a large multicenter cohort of 1016 infants that were hospitalized for acute bronchiolitis, with increased risk for hospitalization in intensive care units and for prolonged LOS [27].

Our main study limitation was the relatively small sample size. Nevertheless, we found a significant difference in 25(OH) vitamin D levels between groups. Additionally, this study had a single-center design, with a specific population; more than half of participants were of Bedouin origin. This population, typically belong to a low socioeconomic status, with large families and overcrowding. They live in poor accommodations/housing conditions, and some Bedouin settlements have limited access to health care. However, we must stress that the 25(OH) vitamin D levels were not significantly different between Bedouin and non-Bedouin subjects; thus, ethnicity was not a bias in this study (Fig. 1). If we compare our results to healthy children living in the region, 25(OH) vitamin D level of 28.6 ng/ml (=71.5 nmol/L) was reported among Jewish children aged 1.5–3 years [28]. 25(OH) vitamin D levels of 99 nmol/L and 72.5 nmol/L were reported at the age of 6 month and 12 months, respectively in an interventional study from our center, with the same population [29]. 25 (OH) Vitamin D level of 31 nmol/L in girls and 22.7 nmol/ L in boys of prepubertal age in Arab population in our country were also reported [30]. Those studies implicate a higher range of vitamin 25 (OH) D levels, even in the same region, among different population of healthy children.

Another limitation is the lack of information on previous medical history of the study population; children in the control group theoretically might have experienced more respiratory tract infections during their first year of life than the bronchiolitis group. The data regarding previous anti asthmatic treatment was collected via questionnaires during the emergency department visits or during the hospitalizations. We assume that the high percentage of anti asthmatic treatment among both groups represent permissive use in the community. The similarity between the groups background may emphasize the role of vitamin D levels as a marker, not just for recurrent or chronic respiratory infections, but also for acute infections.

Additional studies are needed to address the diagnostic and therapeutic implications of the role of vitamin D in respiratory morbidity, particularly in acute bronchiolitis. Future studies should be conducted in multiple centers to include a large number of patients that might better represent the general population.

Our findings suggested that an intervention that could change the vitamin D status in infants might be beneficial. A clear answer to this question could have important implications on public health in the future. Indeed, the prevalence of inadequate vitamin D status is generally high worldwide, particularly among at-risk subpopulation groups. Moreover, in young children, acute bronchiolitis is accompanied by substantial disease burden.

## Conclusion

We found an association between 25(OH) vitamin D deficiency and acute bronchiolitis. This finding suggested that inadequate vitamin D status might serve as a risk factor for acute bronchiolitis. However, findings from this relatively small prospective study warrant further investigation on a larger scale and in different at-risk populations. Those studies might provide a more precise estimate of the potential impact of vitamin D status on acute respiratory infections in children.

## Data Availability

the authors confirm that the data supporting the findings of this study are available within the article and its supplementary materials.
